# The (in)visible cloak: How intersecting stigmas associated with mental and chronic physical disease shape perceptions in a healthcare setting

**DOI:** 10.1111/bjhp.70038

**Published:** 2025-11-27

**Authors:** Edita Fino, Paolo Maria Russo, Maria Ida Gobbini

**Affiliations:** ^1^ Department of Psychology “Renzo Canestrari” Alma Mater Studiorum Università di Bologna Bologna Italy; ^2^ Department of Medical and Surgical Sciences (DIMEC), St. Orsola‐Malpighi Hospital Alma Mater University of Bologna Bologna Italy; ^3^ IRCCS Institute of Neurological Sciences Bologna Italy

**Keywords:** ableism, inclusive healthcare curriculum and medical practice, intersectionality, mental illness stigma, stigma associated with chronic illness

## Abstract

**Objectives:**

Mental illness stigma is widely examined in healthcare, yet less is known about its intersections with the stigma of chronic physical conditions in shaping distinct forms of disadvantage. Here, we investigate whether patients with concurrent mental and physical health conditions are perceived and treated differently by prospective medical doctors.

**Methods:**

Using a mixed‐methods design, preclinical medical students (*N* = 463) evaluated clinical vignettes describing patients with single (i.e., mental or physical chronic disease) and multiple health conditions (i.e., concurrent mental and physical conditions). We assessed emotional reactions, attributions of disease aetiology, caregiving attitudes and meta‐beliefs about patients' disclosure behaviour. Participants were also asked to report as accurately as possible on symptoms presented by each patient in the vignette.

**Results:**

Findings revealed that stigmatized conditions were associated with higher levels of caregiving discomfort, greater disease disclosure reticence and lower levels of symptom recall accuracy. Compared with patients with single conditions, those with concurrent mental and physical illnesses were less likely to receive care, were attributed a lesser tendency to conceal their conditions and had their symptoms recalled less accurately.

**Conclusions:**

Results indicate that when mental and physical illnesses intersect, patients with multiple stigmatized conditions may be differentially perceived in the eyes of medical students and may become (in)visible targets of discrimination in a healthcare setting. We discuss implications for enhancing awareness of social determinants of health and disease for a more representative, responsive and inclusive healthcare curriculum and practice.

**Statement of contribution**

**What is already known on this subject?**

- Some health conditions, like mental illnesses or physical illnesses with visible symptoms, are more stigmatized than others.
- Traditionally, research has adopted a siloed approach, focusing on the negative effects of stigma associated with single diseases.
- The direct effects of mental illness stigma on healthcare delivery and outcomes for patients with mental illness have been widely examined.

**What does this study add?**

- Mental illness stigma operates intersectionally and exacerbates health disparities for patients with co‐morbidity.
- When mental and physical illnesses intersect, patients may become (in)visible targets of discrimination.
- Intersecting stigmas generate complex vulnerabilities and healthcare inequities for people with multiple conditions.

## INTRODUCTION

Illnesses become cumbersome cloaks that seemingly overcome patients and define them as broken. (Corrigan, 2017)

Being affected by a chronic disease can be a profoundly unsettling experience. Beyond the physical and emotional burden of the illness itself, one has to grapple with the realization of being devalued in the eyes of others because of one's poor health condition (Corrigan & Watson, 2002; Crocker & Major, 1989; Goffman, 1963). In cultures where autonomy, productivity, health and beauty are highly valued, illness is often stigmatized as a deviation from social norms—something disgraceful, feared and often concealed. Among stigmatized health conditions, mental illness has garnered a lot of research attention. A substantial body of evidence shows that people with mental illnesses face pervasive and enduring stigmatizing attitudes across social contexts, often stronger than those associated with chronic physical conditions (Link & Stuart, 2017; Pescosolido, 2013). Mental illness stigma is particularly detrimental when manifested in healthcare (Earnshaw & Quinn, 2012; Thornicroft et al., 2007) as it can both explicitly and implicitly affect critical aspects of medical practice such as primary care screening (Carney et al., 2006), routine checks (Roberts et al., 2007), and treatment recommendations (Druss et al., 2000), leading to substandard quality of care for people with mental illness. At the patient level, individuals with mental illnesses often avoid or delay seeking medical care due to fear of judgement, discrimination, or dismissal of their concerns by physicians (Corrigan, 2004). Fear of being stigmatized often leads to nondisclosure of critical health information and prevents individuals from seeking necessary treatments or leveraging resources, such as social support, that could help manage their condition effectively (for reviews see Camacho et al., 2020; Chaudoir & Fisher, 2010). While the effects of stigma associated with mental illness on healthcare outcomes have been widely examined (for a review, see Henderson et al., 2014), much less is known about whether it shapes distinct forms of disadvantage for patients with both mental and chronic physical conditions. In the present study, we address this gap and examine whether patients at the intersection of concurrent mental and physical health conditions are differentially perceived and treated by prospective doctors.

### Stigma and prejudice based on one's health status

Chronic illnesses strike with a double blow, as beside the pain, distress and loss accompanying the disease, they also involve a process of social devaluation that reduces the target in the eyes of others from a whole person to a tainted, discounted, less‐than‐full one (Corrigan & Kosyluk, 2014; Crocker et al., 1998; Goffman, 1963). This phenomenon, commonly described as the social stigma associated with chronic health conditions, is frequently perceived as more challenging than the illness itself and constitutes a substantial component of the overall burden of disease across conditions (WHO, 2011). Research shows that negative stereotypes and prejudice from formal and informal caretakers, including healthcare professionals (also known as provider‐based stigma), may contribute significantly to the burden of disease in people with chronic health conditions (Link & Phelan, 2006).

Also known by the term ‘ableism’, stigma and prejudice around chronic illness originate from commonly shared beliefs about the body's ability, independence and functioning which underscore the tendency to consider healthy and able‐bodied individuals as representatives of the normative social standard, and people with chronic illness and disabilities as largely ‘invisible’ others without a full membership status in society (Bogart & Dunn, 2019; Branco et al., 2019; Campbell, 2009). This aligns with fundamental social psychological functions proposed by Phelan et al. (2008) to explain stigma‐related processes which are: (1) Maintaining social hierarchies (‘keeping people down’), which legitimizes existing inequalities whereby subordinate social groups are considered inferior in terms of basic human qualities like worthiness and value; (2) Enforcing conformity (‘keeping people in’), which reflects societal pressures to comply with social norms that define acceptable behaviour and identity; and (3) Avoidance of perceived threats (‘keeping people away’), which stems from evolutionary pressures to avoid individuals perceived as sick or deformed (Kurzban & Leary, 2001). These processes collectively contribute to the perpetuation of stigma and prejudice towards people with health conditions across various contexts.

### Concealability, controllability and disclosure of stigmatized health conditions

Although rooted in the same processes of social categorization, labeling, stereotyping, negative emotions, social rejection and other forms of discrimination (Major et al., 2013) characteristics associated with stigma and prejudice may vary with the disease (Best & Arseniev‐Koehler, 2023; Pachankis et al., 2018). Whether a stigmatized condition is concealable may influence whether and how one may be exposed to certain forms of direct discrimination (Jones et al., 1984). People tend to hide a stigmatized condition and avoid disclosure for fear of being labelled and not being accepted by others. This choice may not be available for conditions with visible signs of the disease, which are associated with reactions of avoidance and disgust or fear due to aesthetic aspects, often a corollary of the visibility/concealability dimension (Jones et al., 1984; van Beugen et al., 2023). Evidence suggests that our perceptual systems are sensitive to signs of diseases, which may motivate distancing reactions from visible markers (Crandall & Moriarty, 1995) and that such reactions might over‐generalize to aesthetically unappealing, although not contagious, physical traits such as higher weight and missing limbs (Park et al., 2013). For instance, visible cues of psoriasis, an autoimmune skin disease, may elicit disgust reactions and motivate behavioural avoidance, as theorized by disease‐avoidance models (Kurzban & Leary, 2001). Along the same lines, obesity stigma might emerge from similar processes (Park et al., 2007). Because higher‐weight individuals are perceived as having abnormal physical features, they are associated with reactions of disgust and related concerns about disease avoidance (Van Leeuwen et al., 2015), which leads to explicit forms of social rejection.

Concealable conditions such as cancer may also be stigmatized, albeit for different reasons (for reviews, see Chaudoir & Fisher, 2010; Quinn & Earnshaw, 2011). Even though many types of cancers have become chronic, due to improved treatments and diagnostic techniques (Vrinten et al., 2017), cancer remains highly stigmatized because of its strong association with mortality (Penner et al., 2018). Perceptions of control or health‐risk behaviours may contribute to stigma, as in the case of lung cancer, which is stigmatized due to the belief that its primary cause is smoking (Scott et al., 2015). Being able to conceal a stigmatized condition may allow individuals to ‘pass’ as someone without a chronic illness or to downplay the severity of symptoms, protecting them at times from direct forms of discrimination. However, it may also lead to feeling unseen and invalidated by close others and healthcare professionals, who may assume that a disability that cannot be seen is not real (for a review, see Quinn & Earnshaw, 2013). For instance, patients with chronic back pain (Nicola et al., 2021) or functional gastrointestinal disorders like inflammatory bowel syndrome (IBS), often described as ‘illness without disease’ because of the lack of clear pathophysiology, commonly report high levels of stigmatization due to their condition, which has been linked to low treatment adherence, and worse clinical outcomes (for a review see Taft et al., 2017).

### At the intersection of mental and chronic physical illness: the multiple disadvantage

Research on health‐related stigma has greatly focused on mental illness. Although some mental conditions may be concealable (i.e., depression), people with mental illness often refrain from disclosing it to others for fear of being labelled, avoided and discriminated against based on stereotypes that associate mental conditions with negative personal characteristics and dangerousness (Corrigan & Kosyluk, 2014; Jones & Corrigan, 2014; Ziano & Koc, 2023). For instance, people with depression are often viewed as unreliable, uncooperative, incompetent and unable to function autonomously, while they are often blamed for their condition and for not putting enough effort into their recovery, also known as the *weak‐not‐sick* stereotype (Corrigan, 2004; Curcio & Corboy, 2020). Informal caretakers or health professionals may be both targeted by (e.g., associative stigma) and harbour either consciously or unconsciously, the same stereotypes and prejudices found in the general population towards people with mental illness (Fino et al., 2019; Schulze, 2007).

Research indicates that patients with mental health conditions receive poorer‐quality care for both mental and physical health issues. Just as ethnic prejudice contributes to health disparities among minoritized ethnic groups, the stigma around mental illness may drive health disparities in this target group (Link et al., 2018). People with mental illness are at substantially higher risk for overall mortality due to specific causes like heart disease, stroke, diabetes and cancer (Walker et al., 2015). They are also at greater risk for developing poor health behaviours like smoking, obesity and having a sedentary lifestyle. Similarly, they are less likely to receive primary care screening, including screening for cancer (Carney et al., 2006) and routine checks for blood pressure and cholesterol (Roberts et al., 2007), and treatment options (Druss et al., 2000). Therefore, mental illness stigma not only compromises one's chances of receiving appropriate mental healthcare, but it may also significantly curtail the range of healthcare options that would otherwise be available to persons with physical disease, who are not concurrently affected by mental illness.

Recent intersectional frameworks (Jackson‐Best & Edwards, 2018; Stangl et al., 2019; van Brakel et al., 2019) highlight how individuals who become targets of two or more stigmatized health conditions may encounter unique forms of prejudice and discrimination compared with people with single stigmatized or non‐stigmatized conditions (Remedios & Snyder, 2018). Intersectional models capture unique nuances of the disadvantages affecting people who become targets of multiple stigmatized conditions, sustaining that the nature of disadvantage is cumulative (*the double jeopardy perspective*, Beale, 1979) or that multiple devalued conditions may interact to compound the experience of discrimination in unique ways (*the interactive model*, Crenshaw, 1991). In particular, the *intersectional invisibility* model (Purdie‐Vaughns & Eibach, 2008) draws on research on identity prototypicality and group‐based ideologies, suggesting that people with multiple disadvantaged conditions tend to be defined and perceived as non‐prototypical members of their constituent groups, becoming thus socially invisible to others, which may include healthcare workers. Perceived prototypicality is rooted in group‐based ideologies like *ethnocentrism* (the tendency of white dominant majority members to define the standard for society as a whole) and all ‘*ism's*’ that define normative standards in a given context. From this perspective, people with concurrent mental illness and physical health conditions may be defined and perceived as non‐prototypical members (the prototypical member being affected by a single health condition), and will tend to become socially invisible and marginalized members within respective groups (Purdie‐Vaughns & Eibach, 2008). Experiences of invisibility may involve not being remembered, distinguished from others, or recognized for one's contributions (Sesko & Biernat, 2025) and can occur as part of a broader cluster of negative experiences and outcomes among individuals with multiple devalued conditions. Prior research indicates that patients stigmatized across two distinct dimensions—such as chronic illness and minority ethnic status—often have their disease burden and stigma‐coping strategies overlooked by medical students compared with White patients with the same conditions (Fino & Russo, 2025). However, no study to date has investigated healthcare outcomes for patients facing multiple stigmas within the same dimension (health conditions).

### The present study

In the present study, we addressed this gap by examining whether patients with both mental and physical chronic illnesses are differently perceived and approached by prospective medical doctors than those with a single stigmatized condition. In line with previous research (Fino & Russo, 2025), we expected that the more stigmatized conditions (i.e., mental illness and chronic conditions associated with visible manifestations such as psoriasis, obesity) would be associated with more negative emotions (i.e., disgust, fear, pity), with lower levels of caretaking willingness, higher levels of caretaking discomfort and avoidance and a higher tendency to avoid disclosure for fear of negative reactions, compared with concealable and less stigmatized conditions (i.e., irritable bowel disease, cardiomyopathy). In terms of attributions of disease onset, we expected them to align with stereotypical beliefs about controllability of target diseases, with higher personal responsibility being attributed to conditions such as depression, obesity, and lung cancer—Hypothesis 1: Single stigmatization. In addition, in line with the cumulative model of multiple disadvantage (Hypothesis 2a: Multiple stigmatization) we expected that patients with multiple health conditions would be associated with more negative emotions (i.e., disgust, fear, pity), with lower levels of caretaking willingness, higher levels of caretaking discomfort and avoidance and a higher tendency to avoid disclosure for fear of negative reactions, higher attribution of personal responsibility and lower symptom recall accuracy compared with patients with single conditions. However, we also expected that patients with multiple compared with single health conditions may become invisible targets and not equally considered by medical students (Hypothesis 2b: Intersectional invisibility). That is, we expected patients with multiple health conditions to be associated with less negative emotions, higher caretaking propensity, lower caretaking avoidance, lower tendency to avoid disclosure and lower attribution of personal responsibility. As a further test of invisibility, we examined symptom recall for patients with single and multiple health conditions (Sesko & Biernat, 2025). We expected that symptoms of patients with stigmatized conditions (i.e., depression, obesity, psoriasis) would be recalled less accurately (H1: Single stigmatization) and that recall accuracy would be even lower for patients with multiple stigmatized conditions (H2b). Our rationale is based on evidence (Whitty et al., 2020) that healthcare systems, evidence‐based guidelines and medical training tend to be organized along single diseases, which normalizes prototypical representations of patients with single diseases and contributes to prospective doctors feeling less prepared to assess real‐life patients presenting multiple health concerns.

## METHODS

### Participants and procedure

Participants were 463 second‐year medical students (56.2% women, 42.8% men) attending the Medical School of the University of Bologna during 2022–2023. In an experimental, between‐subjects design, participants read and evaluated a series of vignettes describing patients affected by various chronic health conditions. Measures were taken from Fino and Russo (2025) and were originally adapted from Pescosolido (2013), who employed multiple measures to identify core public prejudicial attitudes and behaviours around mental illness across 16 countries. The patient in each vignette was described using gender‐neutral language (i.e., the patient). For one group (*N* = 283), the vignette depicted a patient with a single chronic physical disease, while for the other group (*N* = 180), the patient was additionally described as exhibiting main symptoms of co‐occurring major depression alongside the chronic physical condition. Participants read six vignettes presented in a counterbalanced order and responded to a series of questions on their emotions, thoughts and caretaking behaviour towards the patient. At the end of the survey, participants were asked to write down as many symptoms as they recalled for each patient. All participants provided informed consent and were fully debriefed upon completion of data collection. The study procedure was approved by the Institutional Review Board (IRB; Prot. Nr. 0134185).

### Measures

#### Clinical vignettes

Six vignettes were employed describing patients with a history of depression, psoriasis, obesity, irritable bowel disease (IBS), lung cancer and cardiomyopathy. The vignettes were adapted from Epocrates online materials, were matched in word length, and were used in previous studies (see Fino & Russo, 2025). In the present study, we added a new chronic condition, cardiovascular disease, which is one of the leading causes of death globally (WHO, 2011).

#### Patient health condition manipulation

The patient was presented as either being affected by a single health condition as indicated by the clinical case description (i.e., ‘patient with depression’, ‘patient with psoriasis’, ‘patient with obesity’, ‘patient with irritable bowel disease, IBS’, ‘patient with lung cancer’, ‘patient with cardiomyopathy’) or as being affected by the same condition plus symptoms of concurrent major depression. For each condition, symptoms of major depression were added as follows: ‘…feeling very tired and demoralized and unable to carry out daily functions. Loss of interest in activities that were once pleasurable, including hobbies and relationships’. Because depression was initially presented as a single illness, it was not included among the multiple health conditions. However, since participants in the single‐disease group read and evaluated six different vignettes, we decided to keep the depression vignette in the multiple‐disease group to ensure comparable task demands across groups. To maintain similar word length across vignettes in the multiple‐disease condition, we added symptoms of suicidal ideation to maintain. The data from depression in the multiple‐disease condition were excluded from the main analysis, as they were not relevant to investigating comorbidity, and are instead reported in Table S1.

#### Emotional reactions

To assess emotional reactions associated with target chronic illnesses, we asked participants to indicate the emotion that would best describe their experience when taking care of the patient, from a list including positive and negative emotions (e.g., curiosity, compassion, pity, fear and disgust). Responses were given on a 1 (not at all likely) to 5 (very likely) Likert scale.

#### Attribution of disease aetiology

To evaluate participants' attributions of disease aetiology we asked them to indicate the likelihood that the disease was caused by (a) genetic factors, or (b) unhealthy lifestyle, on a 1 (not at all likely) to 5 (very likely) Likert scale.

#### Caretaking propensity

Three items were used to measure (a) participants' willingness to take care of the patient, (b) the likelihood of avoiding taking care of the patient and (c) the extent to which taking care of the patient would make participants (un)comfortable. Responses were given on a Likert scale from 1 (not at all) to 5 (very much).

#### Meta‐beliefs on patient disclosure behaviour

Participants were asked to indicate how likely or unlikely (1 = not likely at all; 5 = very likely) they thought it was that (a) patients would hide their illness from others and that (b) opening up to others about their disease would elicit negative judgments and attitudes.

#### Symptom recall accuracy

At the end of the survey, we asked participants to write down as many symptoms as possible that they recalled for each patient described in the vignettes. Responses were coded by two independent coders, providing a count of correctly recalled symptoms for each condition, ranging from 0 (no correctly reported symptom) to the highest number of correctly reported symptoms, and incongruencies were rectified by joint decision.

### Statistical analysis

To examine the stigma associated with various diseases, measures of emotional reactions, caretaking propensity, disease origin attributions, and meta‐beliefs on patient disclosure, and symptom recall accuracy were analysed using repeated measures ANOVAs, with the disease as a within‐subjects factor. To further examine multiple stigmatization effects, we conducted six repeated measures ANOVAs on the same measures with (multiple vs. single) health condition as a between‐subjects factor.

## RESULTS

### Emotional reactions

In line with our expectations (H1), emotional reactions typically associated with stigma and avoidance (i.e., disgust, fear and pity) were significantly higher for patients with stigmatized conditions. For instance, the highest level of disgust was reported for psoriasis, whereas fear and pity were highest for lung cancer and depression, which is consistent with stereotypical beliefs about these conditions as being highly threatening and/or having poor prognosis (see Table 1). Notably, positive emotions like compassion and curiosity (*p*s > .05) were the most reported emotions across conditions (all *p*s < .001); see Table 1.

**TABLE 1:** Means (SE) on measures of emotional reactions, caretaking propensity and disease attributions.

	Depression (a)	Obesity (b)	Psoriasis (c)	IBS (d)	Lung cancer (e)	Cardiopathy (f)
Emotional reactions						
Curiosity	2.63 (.06)^bcd^	1.63 (.06)^acdef^	2.14 (.05)^abdef^	1.97 (.05)^abcef^	2.68 (.06)^bcd^	2.65 (.06)^bcd^
Compassion	2.80 (.05)^bcdf^	2.01 (.06)^acef^	2.22 (.05)^abde^	2.05 (.05)^acef^	2.80 (.06)^bcdf^	2.21 (.05)^abde^
Pity	1.82 (.06)^bcdef^	1.35 (.06)^adef^	1.33 (.05)^adef^	1.19 (.05)^abcef^	2.00 (.07)^abcdf^	1.48 (.06)^abcde^
Fear	.62 (.05)^bdf^	.43 (.04)^ace^	.53 (.04)^bdf^	.41 (.04)^ace^	.62 (.05)^bdf^	.42 (.04)^ace^
Disgust	.58 (.05)^cdf^	.62 (.04)^c^	1.07 (.05)^abdef^	.62 (.04)^acef^	.56 (.05)^cd^	.52 (.05)^abcd^
Caretaking variables						
Caretaking willingness	3.71 (.05)^bcdef^	3.24 (.05)^aef^	3.21 (.05)^aef^	3.28 (.05)^aef^	3.85 (.05)^absdf^	4.04 (.04)^abcde^
Caretaking discomfort	1.81 (.04)^bcdef^	1.51 (.04)^adef^	1.58 (.04)^adf^	1.41 (.03)^abcef^	1.62 (.04)^abde^	1.26 (.03)^abcde^
Caretaking avoidance	1.62 (.05)^def^	1.55 (.04)^def^	1.52 (.04)^f^	1.46 (.04)^abf^	1.45 (.04)^abf^	1.32 (.04)^abcde^
Disease origin attributions						
Genetic factors	2.57 (.04)^bcdef^	2.98 (.04)^acf^	3.29 (.05)^abde^	2.97 (.04)^acf^	2.92 (.04)^acf^	3.26 (.04)^abde^
Behavioural factors	3.28 (.05)^bcde^	4.39 (.04)^acdef^	2.27 (.04)^abdef^	3.08 (.04)^abcef^	4.25 (.04)^abcdf^	3.35 (.05)^bcde^

*Note*: Significant differences between conditions are indicated by letters.

Further analysis examining stigma interaction effects showed that disgust levels were significantly higher in the single compared with multiple health conditions, across diseases (all *p*s < .004), and higher levels of fear were reported for patients with psoriasis, obesity and IBS in the single compared with the multiple disease condition (see Table S1). This pattern seems to be more in line with the intersectional invisibility model (hypothesis H2b), which predicts lower levels of negative emotions for multiply stigmatized patients. Instead, higher levels of pity were reported in the multiple compared with the single disease condition for all diseases (all *p*s < .017) except Cardiomyopathy (*p* = .59), which represents the least stigmatized condition in our study. This finding aligns better with the cumulative model of disadvantage (hypothesis H2a), which posits a stronger negative reaction towards multiply stigmatized patients compared with singly stigmatized ones. Interestingly, curiosity was higher for lung cancer and cardiomyopathy (all *p*s < .001), but not significantly different for psoriasis, obesity and IBS, while compassion was higher for obesity (*p* = .003) when patients were affected by concurrent depression (see Table S1). No other significant differences emerged.

### Attributions of disease aetiology

In line with our expectations (H1), results suggested a higher attribution of disease aetiology to unhealthy behaviour compared with genetic factors for depression, obesity and lung cancer (all *p*s < .001), whereas psoriasis was attributed to genetic rather than behavioural causes (*p* < .001). No significant differences were found in attributions of aetiology to genetic or behavioural factors for IBS and cardiomyopathy (*p*s > .07; see Table 1). The analysis examining mental health stigma interactions did not reveal a systematic pattern of results. Attributions of disease to genetic factors were significantly higher for psoriasis (*p* = .001) and IBS (*p* = .016), whereas lung cancer (*p* = .016) and cardiomyopathy (*p* < .001) were more associated with behavioural factors in the single compared with multiple disease conditions. No other differences emerged (for more detailed information, see Table S1).

### Caretaking propensity

**FIGURE 1:**
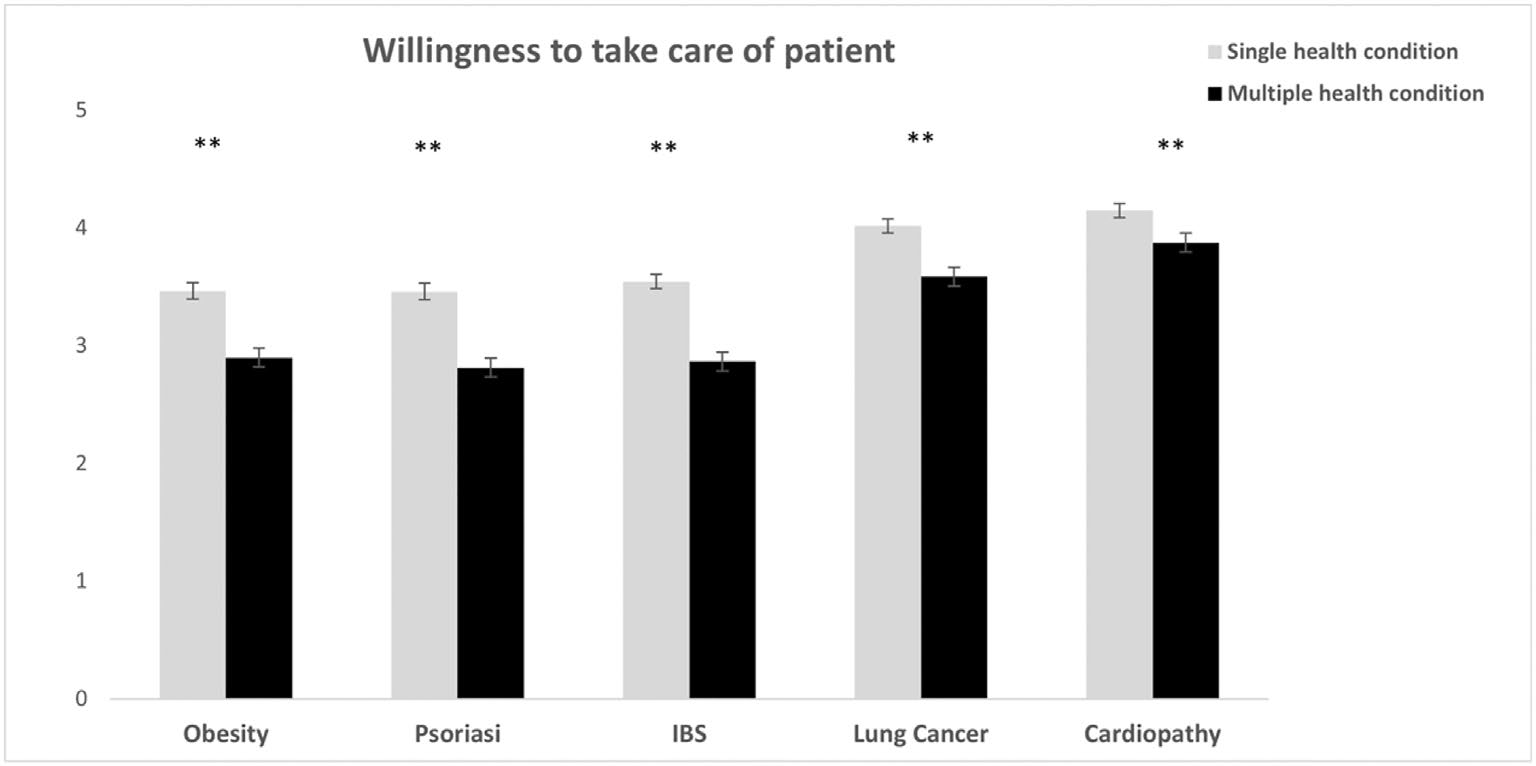
Ratings on caretaking willingness for patients affected by single and multiple health conditions. Error bars represent ±1 SE of the mean. Asterisks indicate significance levels: *p* < .01 (*), *p* < .001 (**).

We expected highly stigmatized conditions to be associated with higher caretaking discomfort and avoidance and a lower caretaking willingness (Hypothesis, H1). In line with our expectations, patients with depression, obesity and psoriasis were associated with higher caretaking avoidance compared with patients with cardiomyopathy, IBS and lung cancer (see Table 1). In addition, the highest levels of willingness to take care were reported for patients with cardiomyopathy (all *p*s < .001), followed by lung cancer (all *p*s < .001) and depression (all *p*s < .001). Further analysis examining stigma interaction effects showed lower levels of caretaking willingness were reported for patients affected by chronic illness and concurrent depression compared with patients with a single disease, across health conditions (see Figure 1). This aligns with the cumulative disadvantage hypothesis (H2a), predicting lower caretaking willingness for multiply stigmatized patients compared with singly stigmatized ones. No other differences emerged in caretaking discomfort and avoidance variables (for more details, see Table S1).

### Meta‐beliefs on disease disclosure

**FIGURE 2:**
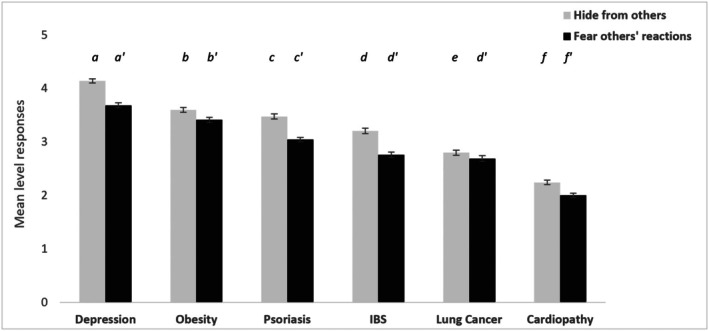
Participants' meta‐beliefs on the (a) patients' hiding their disease from others (grey bars), and (b) reluctance to disclose for fear of others' negative reactions (black bars). Error bars represent ±1 SE of the mean. Bars that do not share a common letter differ significantly from each other at *p* < .001 (Bonferroni‐corrected pairwise comparisons). Letters indicate significant differences across conditions for the ‘Hide from others’ outcome. Prime letters (e.g., a′) indicate corresponding significant differences for the ‘Fear of others' reactions’ outcome.

**FIGURE 3:**
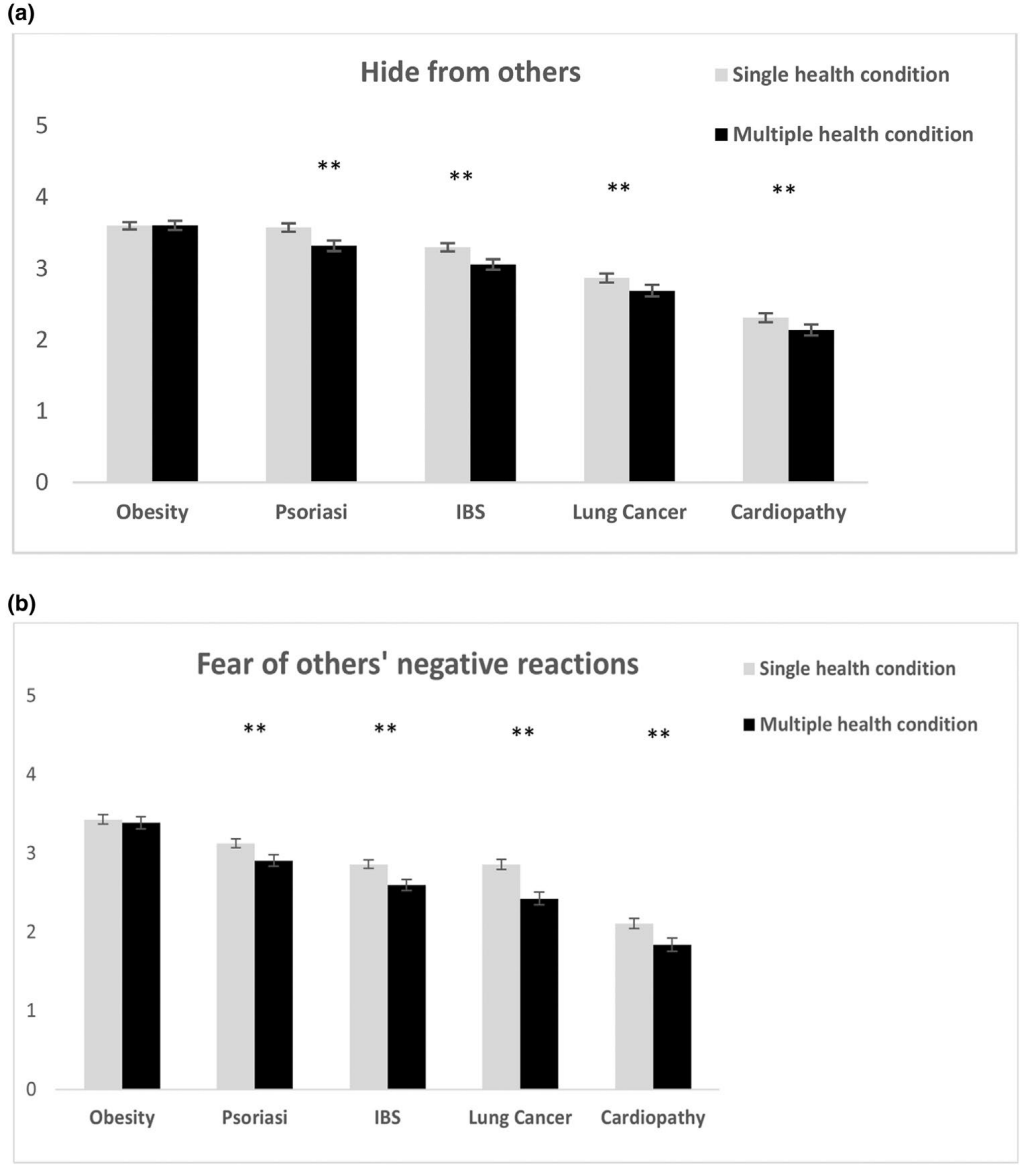
Participants' meta‐beliefs on (a) patients' hiding their disease from others, (b) their being reluctant to disclose for fear of others' negative reactions are shown for single compared with multiple health conditions. Error bars represent ±1 SE of the mean. Asterisks indicate significance levels: *p* < .01 (*), *p* < .001 (**).

When asked to indicate whether the patient would feel embarrassed and tend to hide or avoid disclosure of their condition for fear of others' negative reactions, we expected medical students to associate such behaviours with the more stigmatized conditions (H1). In line with our expectations, results showed a higher tendency to be reluctant to disclose their disease to others and fear of negative consequences when affected by depression, followed by obesity, psoriasis, IBS and lung cancer, with the lowest degree of reticence reported for cardiomyopathy (all *p*s < .0001, except similar levels of fear of negative reactions reported for IBS and lung cancer) (see Figure 2). Furthermore, in line with the intersectional invisibility hypothesis (H2b) participants believed that patients affected by single health conditions tended to hide or avoid discussing their illness with others for fear of negative reactions significantly more compared with patients affected by multiple stigmatized conditions. This effect was consistent across diseases except for obesity (see Figure 3a,b).

### Symptom recall accuracy

**FIGURE 4:**
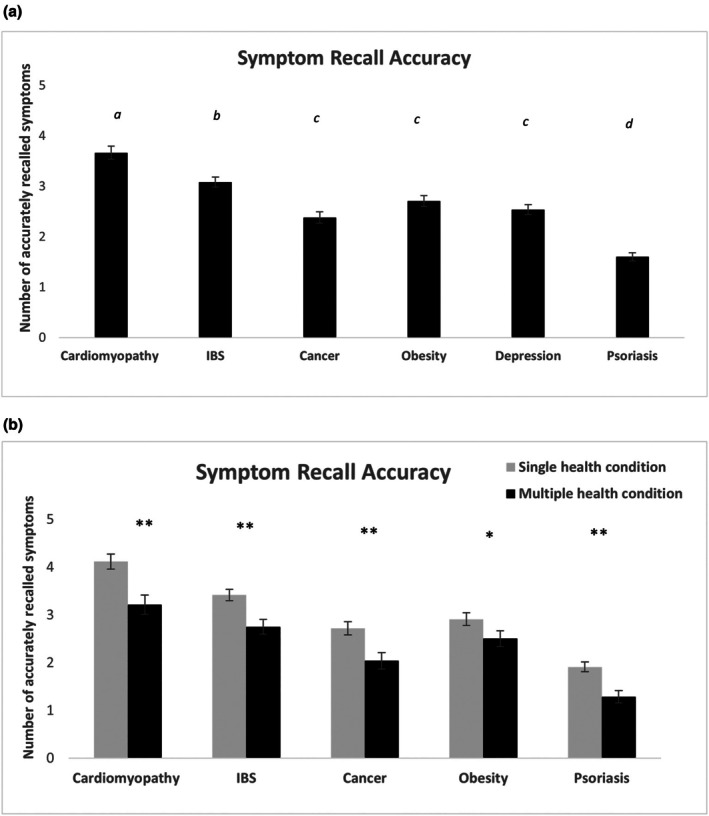
The graph displays the number of accurately recalled symptoms for all patients affected by various diseases (a) and for patients with single and multiple health conditions, respectively (b). Error bars represent ±1 SE of the mean. Bars that do not share a common letter differ significantly from each other at *p* < .001 (Bonferroni‐corrected pairwise comparisons). Error bars represent ±1 SE of the mean. Asterisks indicate significance levels: *p* < .01 (*), *p* < .001 (**).

Results of ANOVAs indicated that symptoms of cardiomyopathy were better recalled compared with all other conditions (all *p*s < .001), followed by IBS (all *p*s < .001), and that psoriasis was the least accurately recalled condition compared with all diseases (all *p*s < .001). Symptoms of depression, obesity and lung cancer were similarly recalled (all *p*s > .115) (see Figure 4a). Findings also showed higher symptom recall accuracy for single compared with multiple stigmatized health conditions, and that this effect was consistent across diseases (all *p*s < .05; see Figure 4b). These results support the invisibility hypothesis, indicating that highly stigmatized conditions are associated with lower recall accuracy (hypothesis H1), and that recall accuracy is even lower for multiple compared with singly stigmatized conditions (hypothesis H2b).

Taken altogether, our results support H1: Single stigmatization hypothesis in terms of emotion reactions, attribution of disease aetiology, caretaking propensity (discomfort and avoidance), meta‐beliefs about disease disclosure and symptom recall. Hypothesis 2a: Cumulative disadvantage was only partially supported by findings on emotion reactions and caretaking willingness, whereas Hypothesis 2b: Intersectional invisibility was partially supported by results on emotion reactions and fully supported by data on meta‐beliefs about patient disclosure, and patient symptom recall.

## DISCUSSION

Moving beyond traditional, siloed approaches that focus on the stigma associated with single diseases and related barriers in care delivery, the present study examined stigma intersections in shaping unique disadvantages for patients with multiple health conditions. Unlike previous research that has examined patients facing multiple stigmas across different dimensions, such as chronic illness and minority ethnic status (Fino & Russo, 2025), our study focused on patients with multiple stigmatized conditions within the same domain: chronic disease. We examined how ableism shapes not only social attitudes but also caretaking propensity towards people with chronic illnesses who are perceived to deviate from the prototypical representation of being healthy and able‐bodied, and are thus considered as less than, or diminished states of being (Campbell, 2009). Our findings indicate that while mental illnesses and physical illnesses with visible symptoms are each highly stigmatized on their own, patients who experience both may be perceived and treated differently by healthcare providers. Such patterns reflect intersecting underlying ableist assumptions that further disadvantage individuals with multiple chronic conditions in a healthcare setting. With an aging population, particularly in Europe, most patients in healthcare settings are likely to present with more than one medical condition. Yet clinical practice and evidence‐based guidelines often remain condition‐specific, offering limited guidance for managing complex comorbidities (Whitty et al., 2020). Our findings corroborated and further extended previous research (Link et al., 2018), providing evidence that mental illness stigma operates in an intersectional way, exacerbating disparities in perceptions and likely outcomes for individuals with chronic illness and depression co‐morbidity in a healthcare setting. Importantly, results point to the role of intersecting stigmas in generating complex vulnerabilities and healthcare inequities for people affected by multiple stigmatized conditions.

Consistent with previous studies (Fino et al., 2021; Fino & Russo, 2025; Fiske, 2012; Kurzban & Leary, 2001), higher levels of disgust were reported for psoriasis, an autoimmune skin disease characterized by visible manifestations. This is in line with disease‐avoidance models, which suggest that visible conditions can activate responses of disgust and motivate avoidance behaviours (Kurzban & Leary, 2001). In addition, fear and pity were most frequently reported for depression and lung cancer, reflecting stereotypical perceptions of these conditions as highly threatening and as having poor prognosis. In line with established stereotypes about the perceived responsibility of patients for their health, attributions of disease aetiology for depression, obesity and lung cancer were linked to behavioural rather than genetic factors compared with the other conditions. This finding coheres with previous research indicating that these conditions are often perceived as controllable, leading to blame and attributions of individual responsibility (Jones et al., 1984). In terms of caretaking propensity, findings support our first hypothesis (H1) predicting higher caretaking discomfort and avoidance for stigmatized conditions. Indeed, participants reported more caretaking discomfort for depression compared with all other conditions, followed by obesity and psoriasis, with the lowest levels reported for cardiomyopathy and IBS. Also, caretaking avoidance was higher for depression, obesity and psoriasis compared with cardiomyopathy, IBS and lung cancer. These findings are consistent with previous literature showing that people with mental illness and conditions with visible signs are more stigmatized compared with conditions whose signs are not immediately noticeable (Best & Arseniev‐Koehler, 2023; Fino & Russo, 2025; Goffman, 1963; Jones et al., 1984). The fact that hypothesis 1 was not supported for caretaking willingness may be explained by social desirability bias, which is in line with previous findings from Fino and Russo (2025).

Our first hypothesis was further corroborated by the more indirect measures we used to minimize the risk of social desirability bias that might have played a role when participants provided more explicit responses (i.e., caretaking propensity questions). Participants' meta‐beliefs about disease disclosure suggested that patients with depression, obesity and psoriasis were perceived as experiencing higher reluctance to disclose their conditions due to fear of negative consequences. In contrast, cardiomyopathy, identified as the least stigmatized condition in the study, was associated with the lowest levels of perceived negative consequences related to disease disclosure. Particularly, the symptom recall task, which was used as an invisibility measure, yielded a similar pattern of results. Psoriasis was the least accurately recalled condition, followed by depression, obesity and lung cancer, which exhibited similarly low levels of recall accuracy. In contrast, cardiomyopathy and IBS were recalled more accurately compared with the other conditions. These findings are in line with previous research (Sesko & Biernat, 2025) and support our hypothesis (H1) that highly stigmatized conditions are associated with reduced recall accuracy, which may be attributed to prejudicial attitudes that may affect information processing, retention and recall processes. These results highlight that biases associated with single diseases, particularly mental illness and chronic physical conditions with visible manifestations, influence emotional reactions, attitudes and caregiving behaviours among prospective medical doctors and affect recall processes that may likely interfere with medical decision‐making.

The main aim of our study was to investigate whether being concurrently affected by a physical and mental health condition makes one more prone to experiencing multiple disadvantages in a healthcare setting. Given the high prevalence of depression as a comorbid condition in chronic illnesses (Thom et al., 2019), we tested the hypothesis (H2) examining whether patients with chronic physical illnesses and concurrent depressive symptoms would be subject to unique disadvantages compared with those with a single stigmatized condition. Findings on emotional reactions revealed a complex pattern of significant differences, partly in line with our expectations. Unlike previous studies (Fino & Russo, 2025) showing that ethnic cues can amplify negative emotions of disgust and fear, particularly for visible or physically manifested conditions, our findings revealed that the presence of multiple illnesses, especially when one is a mental disease, can attenuate certain negative emotions and enhance compassion or curiosity. Here, the key pattern is that pity tends to increase in multiple‐disease scenarios, which would be more in line with the cumulative disadvantage hypothesis (H2a), while disgust and fear are higher in single medical conditions, findings that partly align with the intersectional invisibility hypothesis (H2b). The reduced disgust and fear in the multiple‐disease context could reflect a shift in perception from a stigmatizing lens towards a more compassion‐oriented or medicalized framing of the patient. Interestingly, positive emotions such as curiosity also emerge in specific multiple‐disease contexts, indicating that comorbidity can sometimes evoke engagement rather than avoidance. These results suggest that affective reactions towards patients with multiple health conditions are complex and often ambivalent, characterized by both positive and negative emotions. This aligns with contemporary prejudice theories and research (Dovidio et al., 2017), suggesting that individuals may hold conflicting feelings about socially devalued people or groups. Indeed, ambivalent reactions to chronic illness are more common than usually assumed (for a review, see Fiske, 2012). It has been shown that people may have intense yet conflicting feelings towards individuals with mental illness, who might be perceived as both deviant and disadvantaged (Crocker et al., 1998). The finding that pity—a more ambivalent emotional reaction compared with fear and disgust, was higher in the multiple compared with the single disease condition, further supports this tendency suggesting that ambivalent emotional reactions may be more relevant in the context of multimorbidity, whereas negative emotions such as fear and disgust, more traditionally linked with stigma and avoidance, may be more prototypically characteristic of singly stigmatized conditions.

Our findings on caretaking willingness further support the multiple disadvantage hypothesis (H2a). Compared with patients with single conditions, participants' willingness to take care of those affected by concurrent depression and physical chronic illness was significantly lower across diseases. This indicates that at the intersection of stigma associated with mental illness and stigma associated with chronic disease, patients are less likely to be taken care of by health professionals. These results confirm existing evidence showing that mental illness stigma may contribute to suboptimal care for patients affected by mental and physical health conditions (Earnshaw & Quinn, 2012; Thornicroft et al., 2007). To our knowledge, this is the first report suggesting that stigma associated with mental illness operates in an intersectional way and exacerbates disparities in health outcomes for persons with multiple diseases. The evidence that patients affected by multiple conditions are differentially perceived and cared for by medical students was further corroborated by the more indirect measures used in our study. For instance, participants believed that patients affected by multiple compared with single stigmatized diseases would be less likely to hide or avoid discussing their illness with others for fear of negative reactions, an effect that was consistent across diseases except for obesity. These findings are in line with our hypothesis (H2b) and seem to extend previous research on the marginalization and social invisibility experiences reported by individuals with multiple stigmatized statuses (Fino & Russo, 2025; Purdie‐Vaughns & Eibach, 2008), suggesting that medical students might fail to equally consider stigma‐related concerns in patients with multi‐morbidity. The lack of significant differences for obesity when considered singly or with depression in comorbidity might be related to the fact that prototypical conceptions of people with depression and obesity are similar in terms of psychological, social and physical traits (Ziano & Koc, 2023). Furthermore, symptom recall accuracy, which was an invisibility measure in our study, was significantly lower for patients with multiple compared with single health conditions, suggesting that prejudicial attitudes towards mental illness may uniquely shape perception, retention and recall of clinical information in healthcare settings, further disadvantaging these patients. Our findings are in line with and expand upon previous research (Fino & Russo, 2025), indicating that individuals with multiple health conditions are at increased risk of experiencing unique forms of prejudice and discrimination in a healthcare setting.

This perspective is consistent with a growing body of evidence (Whitty et al., 2020) highlighting how medical education and training are predominantly structured around the management of single diseases. It should be noted that a key limitation of this study is its reliance on hypothetical vignettes, which capture participants' intended attitudes and reactions rather than observed behaviours in real clinical settings. While vignettes are a useful tool for exploring sensitive topics, they may not fully reflect how medical students would act in practice. Future research involving interactions with actual patients may be necessary to assess how these attitudes influence clinical decision‐making. Moreover, we manipulated concurrent depression implicitly by adding significant symptoms in the presentation of patients in each vignette; hence, participants were not aware of a concurrent depression diagnosis. Future studies should test stigma‐related effects on caretaking attitudes through a more explicit manipulation of concurrent mental or physical health conditions. Furthermore, attention should be paid to evaluating the effects of exposure, familiarity and quality of contact with people affected by different diseases, as these factors may moderate stigma and prejudice associated with health conditions in prospective doctors. It is important to note that this study focused specifically on stigma related to mental and physical chronic disease and did not examine other forms of intersecting discrimination, such as racism and sexism. These intersecting forms of discrimination may compound the effects of mental health stigma, particularly for women patients and individuals from marginalized backgrounds (Fino & Russo, 2025). Future studies should aim to adopt an intersectional framework that captures the complex interplay of multiple identities and forms of bias in medical settings.

## CONCLUSION

Our findings provide insight into how health‐related stigmas operate in the context of single and multiple chronic conditions, especially when mental and physical illnesses intersect. We show that mental illness stigma, while being particularly strong and pervasive when considered by itself, may play an even more insidious role when interacting with the stigma associated with chronic conditions, further exacerbating healthcare inequalities for people with multiple health concerns. We highlight the complexities of intersecting stigmas, where the simultaneous presence of mental and physical health conditions in patients may be perceived as an (in)visible cloak, exacerbating existing biases and potentially leading to compounded disadvantages in healthcare settings. By focusing on the perspective of healthcare professionals and using both direct and indirect measures of attitudes towards patients with multiple chronic conditions, we contribute to a more nuanced picture of how ableism undermines the ethical principles of equity, dignity and patient‐centered care, and shapes the manifold manifestations of prejudice in a healthcare setting.

## AUTHOR CONTRIBUTIONS

**Edita Fino:** Conceptualization; investigation; writing – original draft; writing – review and editing; methodology; data curation; formal analysis. **Paolo Maria Russo:** Conceptualization; investigation; writing – review and editing; methodology; formal analysis; supervision. **Maria Ida Gobbini:** Conceptualization; investigation; writing – review and editing; supervision; formal analysis.

## Supporting information


Table S1.

## Data Availability

The data that support the findings of this study are available from the corresponding author upon reasonable request.
