# Supplementary material for: The (in)visible cloak: How intersecting stigmas associated with mental and chronic physical disease shape perceptions in a healthcare setting
Source: Br J Health Psychol. 2025 Nov 27;31(1):e70038. doi: 10.1111/bjhp.70038 (PMC12658469; doi:10.1111/bjhp.70038)
Supplement: Supplementary file 1 — Table S1. [file BJHP-31-0-s001.docx]

**Table S1**. Means and Standard Errors (SE) on measures of emotional reactions, caretaking propensity, and disease attributions per single and multiple health conditions.

|  | |  |  |  |  |  |
| --- | --- | --- | --- | --- | --- | --- |
| a. | **Single Health Condition** | | | | | |
|  | *Depression* | *Obesity* | *Psoriasis* | *IBS* | *Lung cancer* | *Cardiopathy* |
| *Emotional Reactions* |  |  |  |  |  |  |
| Curiosity | **2.54 (0.07)** | 1.64 (0.07) | 2.07 (0.07) | 1.92 (0.07) | **2.56 (0.07)** | **2.45 (0.07)** |
| Compassion | 2.76 (0.07) | **1.87 (0.07)** | 2.16 (0.07) | 2.07 (0.07) | 2.74 (0.07) | 2.20 (0.07) |
| Pity | **1.66 (0.08)** | **1.14 (0.07)** | **1.16 (0.07)** | **1.05 (0.07)** | **1.87 (0.08)** | 1.45 (0.07) |
| Fear | 0.66 (0.06) | **0.58 (0.05)** | **0.68 (0.05)** | **0.57 (0.05)** | 0.64 (0.06) | 0.47 (0.05) |
| Disgust | **0.90 (0.06)** | **0.72 (0.06)** | **1.32 (0.06)** | **1.04 (0.06)** | **0.84 (0.06)** | **0.79 (0.06)** |
| *Caretaking variables* |  |  |  |  |  |  |
| Caretaking Willingness | **3.83 (0.07)** | **3.46 (0.07)** | **3.46 (0.06)** | **3.54 (0.06)** | **4.02 (0.06)** | **4.15 (0.05)** |
| Caretaking Discomfort | 1.87 (0.06) | 1.51 (0.05) | 1.51 (0.05) | 1.36 (0.04) | 1.61 (0.05) | **1.31 (0.04)** |
| Caretaking Avoidance | 1.69 (0.06) | 1.57 (0.06) | 1.54 (0.05) | 1.48 (0.04) | 1.48 (0.05) | 1.33 (0.05) |
| *Disease Origin Attributions* | |  |  |  |  |  |
| Genetic factors | 2.60 (0.05) | 2.96 (0.05) | **3.39 (0.05)** | **3.05 (0.05)** | 2.95 (0.05) | 3.31 (0.05) |
| Behavioral factors | 3.35 (0.06) | 4.44 (0.04) | 2.29 (0.05) | 3.11 (0.05) | **4.31 (0.05)** | **3.57 (0.05)** |
| b. | **Multiple Health Condition** | | | | | |
|  | *Depression** | *Obesity* | *Psoriasis* | *IBS* | *Lung cancer* | *Cardiopathy* |
| *Emotional Reactions* |  |  |  |  |  |  |
| Curiosity | **2.77 (0.09)** | 1.62 (0.09) | 2.14 (0.09) | 2.05 (0.09) | **2.86 (0.09)** | **2.97 (0.09)** |
| Compassion | 2.87 (0.09) | **2.21 (0.09)** | 2.28 (0.09) | 2.02 (0.09) | 2.89 (0.09) | 2.23 (0.09) |
| Pity | **2.08 (0.10)** | **1.68 (0.09)** | **1.60 (0.09)** | **1.41 (0.09)** | **2.20 (0.10)** | 1.52 (0.09) |
| Fear | 0.56 (0.08) | **0.19 (0.08)** | **0.29 (0.08)** | **0.17 (0.06)** | 0.60 (0.08) | 0.34 (0.07) |
| Disgust | **0.07 (0.08)** | **0.45 (0.07)** | **0.65 (0.08)** | **0.53 (0.08)** | **0.09 (0.08)** | **0.06 (0.08)** |
| *Caretaking variables* |  |  |  |  |  |  |
| Caretaking Willingness | **3.53 (0.08)** | **2.90 (0.08)** | **2.81 (0.08)** | **2.86 (0.08)** | **3.58 (0.08)** | **3.87 (0.07)** |
| Caretaking Discomfort | 1.73 (0.07) | 1.50 (0.06) | 1.54 (0.05) | 1.48 (0.06) | 1.62 (0.07) | **1.19 (0.05)** |
| Caretaking Avoidance | 1.53 (0.08) | 1.53 (0.07) | 1.50 (0.06) | 1.41 (0.06) | 1.39 (0.06) | 1.29 (0.06) |
| *Disease Origin Attributions* | |  |  |  |  |  |
| Genetic factors | 2.52 (0.07) | 3.03 (0.07) | **3.14 (0.06)** | **2.86 (0.07)** | 2.88 (0.06) | 3.20 (0.06) |
| Behavioral factors | 3.20 (0.07) | 4.31 (0.06) | 2.26 (0.07) | 3.04 (0.07) | **4.14 (0.06)** | **2.99 (0.07)** |

*Note*: Values in bold indicate significant differences between single vs. multiple health conditions.

*Information on Depression was included here for transparency but was excluded from the main analysis.
